# Risk Factors for Glaucoma and Ocular Hypertension and Post-Transplant Complications in Keratoconus: A Multivariable Analysis

**DOI:** 10.3390/jcm13185407

**Published:** 2024-09-12

**Authors:** Magdalena Nandzik, Adam Wylęgała, Dominika Szkodny, Ewa Wróblewska-Czajka, Edward Wylęgała, Bogusława Orzechowska-Wylęgała

**Affiliations:** 1 Department of Ophthalmology, Faculty of Medical Sciences, Zabrze Medical University of Silesia, 40-760 Katowice, Poland; 2 Department of Ophthalmology, District Railway Hospital in Katowice, Medical University of Silesia, Panewnicka 65, 40-760 Katowice, Poland; 3 Department of Pediatric Otolaryngology, Head and Neck Surgery, Medical University of Silesia (SUM), 40-055 Katowice, Poland

**Keywords:** keratoconus, glaucoma, risk factors, keratoplasty, keratoplasty complications, ocular hypertension

## Abstract

**Background**: The purpose of this study was to investigate the risk factors for glaucoma in patients with keratoconus following keratoplasty and to identify potential factors that could affect post-transplant complications. **Methods**: A retrospective analysis was conducted on the medical records of 192 keratoconus patients who underwent keratoplasty. Data on treatment history, perioperative information, tissue bank data, postoperative regimens, complications, and infections were collected and analyzed. Statistical analysis was performed to identify risk factors associated with glaucoma and transplant complications. **Results**: There was a 41.6% incidence of glaucoma (high IOP) (*p* < 0.001), with the median time to glaucoma development being 314 ± 67 days post-transplant. A significant number of patients required surgical intervention, accounting for 48.05%. Our analysis revealed a 14% graft failure rate. On univariable Cox proportional hazard analysis, the following factors demonstrated statistically significant associations with the risk of glaucoma after transplantation: donor endothelial cell density, the use of a single continuous suture type, and the surgeon (performing the surgery). For many variables, the only factor that remained significant was the surgeon. Among the factors analyzed for risks of failure post-transplantation, significant associations were observed for the donor age, the time from harvest to transplant, and the surgeon. In the multivariable analysis, donor age emerged as a significant predictor of post-transplant complications. **Conclusions**: Risk factors such as donor endothelial cell density, suture type, surgeon, host and donor size, and host gender were found to increase the risk of developing glaucoma post-keratoplasty. Surgeon type was identified as a significant risk factor, while donor age was predictive of post-transplant complications.

## 1. Introduction

Keratoconus is the predominant corneal ectatic disorder, characterized by gradual corneal thinning leading to irregular astigmatism and myopia [1]. Although initially considered non-inflammatory, recent findings contradict this view [2,3]. The global prevalence of keratoconus is estimated to affect approximately 1 in every 375 to 2000 individuals [4]. Studies conducted on a national level in European countries, such as France and Ireland, have indicated that keratoconus is the primary reason for keratoplasty, representing approximately 19% of cases [5,6,7]. In a study conducted at our center, we found that keratoconus is the second most common indication for keratoplasty, accounting for 18.07% of all 758 corneal transplants [7].

In recent years, a decline in the frequency of keratoplasty procedures for keratoconus has been observed due to the adoption of corneal cross-linking techniques [8]. However, this does not change the fact that a large proportion of patients still require or will require a corneal transplant. Glaucoma is a potentially severe complication that can occur after keratoplasty, leading to the loss of visual potential in the eye due to irreversible damage to the optic nerve [9]. Glaucoma is also a significant complication following corneal transplantation and is a common cause of graft failure, as well as the main cause of vision loss after keratoplasty [10]. According to various sources, the incidence of glaucoma after corneal transplantation is estimated to be between 5.3% [11] and 60% [12]. An extensive systematic review and meta-analysis, including thirty studies, showed that the average incidence of glaucoma after keratoplasty was 21.5% [13]. This study also identified risk factors for glaucoma after transplantation, with strong risk factors being preexisting glaucoma and aphakia, while modest predictors included pseudophakia, regrafting, and trauma. Authors of another comprehensive systematic review identified the following conditions as likely associated with an increased risk of ocular hypertension after keratoplasty: glaucoma in the contralateral eye, an indication of bullous keratopathy, African American descent, preoperative treatment with cyclosporine or olopatadine 0.1%, postoperative treatment with prednisolone acetate 1%, and combined surgery [14]. Risk factors for the rejection of PK include the following [15]: preoperative inflammation, corneal neovascularization (>2 quadrants), young recipient age, iris synechiae to the graft margin, large grafts, a history of inflammatory disease, prior ocular surgery, loose sutures before graft rejection, and prior use of glaucoma medications or surgery.

Risk factors for glaucoma and ocular hypertension after keratoplasty have been described in many research studies [16,17,18,19,20,21,22]. A common feature among these studies is that the factors are determined for the entire group of indications for transplantation. Since we could not find an article on this topic specifically dedicated to keratoconus, we aimed to study the risk factors for glaucoma in patients with keratoconus after keratoplasty. Our primary objective was to use robust statistical methods to explore whether these variables might influence the development of glaucoma ocular hypertension in patients with keratoconus who underwent corneal transplantation. Moreover, having data on the complications that occurred in patients, we sought to identify risk factors that could potentially influence post-transplant complications, such as rejection.

## 2. Materials and Methods

### 2.1. Methodology

A detailed retrospective analysis was conducted between July 2014 and August 2023 at the Department of Ophthalmology of the District Railway Hospital in Katowice, Poland. We reviewed the medical records of patients who were systematically followed according to a predefined schedule. During each follow-up visit, intraocular pressure (IOP) was routinely assessed. If elevated IOP was detected, appropriate pharmacological treatment was promptly initiated. In addition to IOP measurement, other diagnostic tests, such as optical coherence tomography (OCT) of the retinal nerve fiber layer (RNFL) and visual field testing, were performed whenever possible. These tests were crucial for determining disease progression, evaluating treatment efficacy, and considering the need for surgical intervention.

The analysis included a comprehensive assessment of the medical and surgical histories of patients with keratoconus who underwent keratoplasty. The patients’ medical records were reviewed for age, gender, diagnosis for which keratoplasty was performed, and preoperative visual acuity measurements, as well as the use of antiglaucoma medications and surgical procedures. We analyzed the time after transplantation when antiglaucoma treatment was initiated and the type of treatment used. The perioperative risk factors for glaucoma and ocular hypertension analyzed included the type of graft, use of laser during surgery (laser-assisted trepanation), graft size (size of the host), donor size, type of suturing, suture thickness, and the surgeon performing the procedure. The tissue eye bank risk factors for glaucoma analyzed included data such as type of donation, donor gender, donor age, time from death to donation, time from donation to transplantation, and the number of donor endothelial cells. All postoperative regimens, follow-up complications, and infections were analyzed and included.

To assess the risk of complications after corneal transplantation in patients with keratoconus, a multivariate analysis was performed based on the collected data. To verify the impact of the transplant on the quality of vision, we decided to analyze the best-corrected visual acuity (BCVA) before and one year after the corneal transplant.

However, the retrospective nature of our study and the specific challenges associated with post-keratoplasty patients, such as poor visualization and measurement inaccuracies, limited our ability to consistently report on OCT RNFL and visual field outcomes. Performing these tests on this patient population was often difficult due to corneal irregularities and opacities resulting from the keratoplasty procedure, which can compromise the accuracy and reliability of the results.

Given these limitations, we chose to focus our analysis on the treatment strategies used for managing elevated IOP in our study population. We adopted this approach because treatment patterns—such as the escalation from monotherapy to combination therapy or the decision to proceed with surgical intervention—can provide indirect but meaningful insights into disease progression and treatment response. Specifically, we reported the number of patients who were successfully managed with monotherapy, those requiring the addition of a second or third medication, and those who eventually needed surgical intervention despite maximal pharmacological therapy.

This treatment-based analysis allows us to draw conclusions about the effectiveness of various therapeutic approaches in this unique patient population. We believe that our findings offer valuable information on the practical management of elevated IOP in post-keratoplasty patients, even in the absence of consistent OCT RNFL and visual field data.

### 2.2. Glaucoma Definition

Glaucoma is a chronic, progressive optic neuropathy characterized by specific morphological changes in the optic nerve and retinal nerve fiber layer, occurring in the absence of other ocular diseases or congenital defects. Defining glaucoma after keratoplasty presents several challenges [23]. Preoperative assessment of the optic nerve, visual field, and intraocular pressure (IOP) can be difficult due to corneal opacity. Postoperative evaluation may also be problematic due to factors such as high astigmatism and reduced corneal transparency. Post-keratoplasty glaucoma is defined as a sustained elevated IOP of ≥22 mmHg at various time points, leading to the introduction of antiglaucoma medication or surgical intervention, with or without associated visual field loss or optic nerve head changes [24,25,26]. This is the definition of glaucoma we used in our study.

### 2.3. Statistical Analysis

Statistical analyses were performed using IBM SPSS Statistics for Microsoft, version 28.0 (IBM Inc., Armonk, NY, USA), and Statistica 13.3 (Tibco, Palo Alto, CA, USA). The data health check function was used to identify missing data, outliers, and sparse data. Descriptive statistics summarized demographic information and cell characteristics. The McNemar test was used to compare glaucoma status before and after surgery in the same individuals. Kaplan–Meier survival analysis was applied to calculate the time to glaucoma onset and graft failure. Cox proportional hazards analysis was used to assess factors associated with the time to glaucoma onset and graft failure. *p*-values less than 0.05 were considered statistically significant.

## 3. Results

### 3.1. Demographic Data and Clinical Characteristics of the Study Population

Among the study participants, 48 (25%) were female and 144 (75%) were male, totaling 192 individuals. The mean age of the participants was 48.39 years (SD = 17.626), ranging from 21 to 92 years. The average follow-up duration was 51.75 months (SD = 20.753), with a range from 12 to 108 months.

### 3.2. Occurrence of Glaucoma before and after Corneal Transplantation and Treatment Strategies

**Figure 1:**
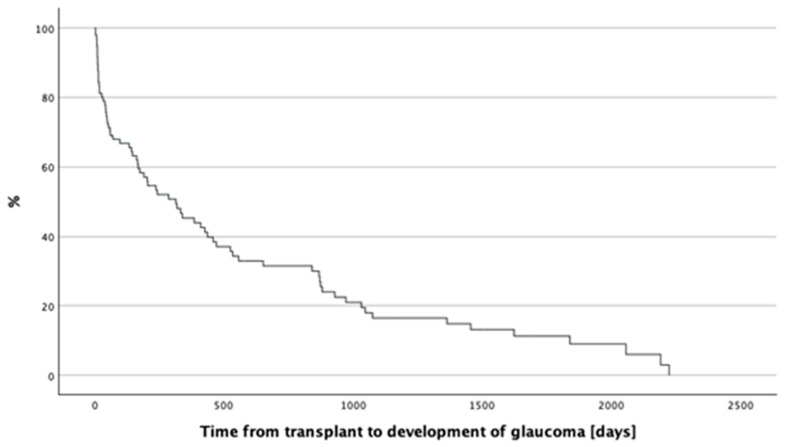
Kaplan–Meier survival curve depicting time to development of glaucoma.

Of the total 192 patients, 185 patients (96.35%) had no history of glaucoma before the transplant, while 7 patients (3.65%) did. Among the 185 patients without a history of glaucoma prior to the transplant, 108 (58.40%) did not develop new cases of glaucoma after the transplant, whereas 77 patients (41.6%) did (*p* < 0.001). The median time to develop glaucoma post-transplant was 314 ± 67 days. The analysis revealed that 26 patients (33.77%) required therapy with two antiglaucoma eye drops, 43 patients (55.84%) required monotherapy, and 8 patients (10.39%) needed treatment with three different antiglaucoma agents. Furthermore, 37 patients (48.05%) required surgical intervention. Figure 1 presents the Kaplan–Meier survival curve illustrating the time to glaucoma development.

### 3.3. Distribution of Transplant Types

The distribution of transplant types in the study cohort was as follows: penetrating keratoplasty (PK)—159 (82.81%), triple—20 (10.42%), Descemet’s stripping automated endothelial keratoplasty (DSAEK)—3 (1.56%), anterior lamellar keratoplasty (ALK)—2 (1.04%), ALK big bubble—4 (2.08%), ALK deep anterior lamellar keratoplasty (DALK)—1 (0.52%), and triple DSAEK—3 (1.56%). Regarding laser usage during surgery, 154 patients (80.21%) underwent surgery without a laser, 16 (8.33%) underwent femtosecond laser-assisted surgery, and 22 (11.46%) underwent excimer laser-assisted surgery. Additionally, various surgeons performed the transplantations: Surgeon 1 performed 77 surgeries (40.10%), Surgeon 2 performed 36 (18.75%), Surgeon 3 performed 34 (17.71%), Surgeon 4 performed 4 (2.08%), Surgeon 5 performed 33 (17.19%), and Surgeon 6 performed 3 (1.56%).

### 3.4. Potential Risk Factors of Glaucoma

Potential risk factors of glaucoma and ocular hypertension were examined using a univariable Cox proportional hazards model. Several factors demonstrated statistically significant associations (*p* < 0.05) with the risk of glaucoma after transplantation. Donor endothelial cell density (ECD/mm^2^) was associated with an increased risk of glaucoma (HR = 0.999; 95% CI: 0.998 to 1.000; *p* = 0.049). The use of a single continuous suture was associated with a significantly higher risk of glaucoma (HR = 4.055; 95% CI: 1.503 to 10.936; *p* = 0.006). Furthermore, the surgeon performing the surgery significantly influenced the risk of glaucoma (HR = 2.058; 95% CI: 1.410 to 3.004; *p* < 0.001). Additionally, a decrease in the size of the host was significantly associated with a higher risk of glaucoma (HR = 0.001; 95% CI: 0.000 to 0.487; *p* = 0.029). Similarly, a decrease in the size of the donor was significantly associated with a higher risk of glaucoma (HR = 0.003; 95% CI: 0.000 to 0.712; *p* = 0.037). Lastly, female hosts exhibited a significantly lower risk of glaucoma compared to male hosts (HR = 0.292; 95% CI: −0.099 to 0.863; *p* = 0.026).

For many variables, the only factor that remained significant was the surgeons performing the surgery. Surgeon 2 and Surgeon 3 showed a significant decrease in glaucoma risk (HR = 0.35, 95% CI: 0.18 to 0.68, *p* = 0.002; HR = 0.40, 95% CI: 0.20 to 0.81, *p* = 0.011, respectively). Additionally, Surgeon 4 demonstrated a marginally significant association with glaucoma risk (HR = 0.45, 95% CI: 0.20 to 1.02, *p* = 0.055). Results for the Cox proportional hazard of glaucoma after transplantation are presented in table (Table 1).

### 3.5. Risks of Failure

Within our group, we observed 27 graft failures, accounting for 14.06% of the cases. The median time to graft failure was 364 ± 15 days. Among the complications, the most common were rejection (4.69%), edema (2.08%), decompensation (1.56%), persistent erosion (1.04%), and trauma (1.04%) as shown in Table 2.

**Figure 2:**
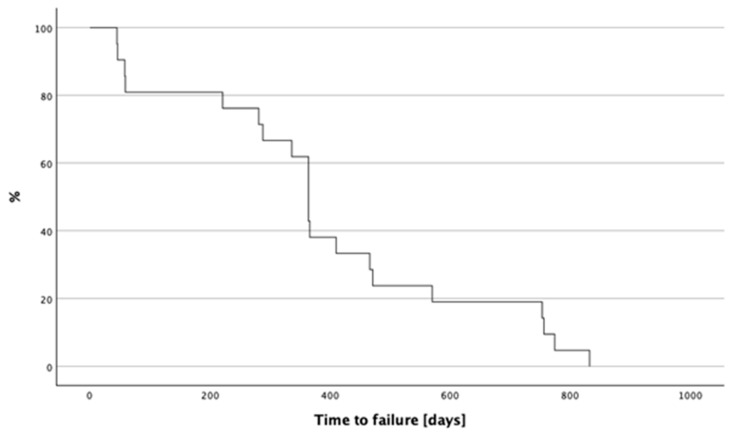
Kaplan–Meier survival curve depicting time to graft failure.

Among the factors analyzed for risks of failure post-transplantation, significant associations (*p* < 0.1) were observed for donor age (HR = 1.114; 95% CI: 0.979 to 1.268, *p* = 0.04), time from harvest to transplant (HR = 0.605; 95% CI: 0.343 to 1.067, *p* = 0.083), and surgeon (HR = 0.265; 95% CI: 0.093 to 0.759, *p* = 0.013). In the multivariable analysis, donor age emerged as a significant predictor of the post-transplant complications (HR = 1.032, SE = 0.013, Wald = 5.681, df = 1, *p* = 0.017). The chart below (Figure 2) shows a Kaplan–Meier survival curve depicting time to graft failure.

Among the factors analyzed for risks of failure post-transplantation, significant associations (*p* < 0.1) were observed for donor age (HR = 1.114; 95% CI: 0.979 to 1.268, *p* = 0.04), time from harvest to transplant (HR = 0.605; 95% CI: 0.343 to 1.067, *p* = 0.083), and surgeon (HR = 0.265; 95% CI: 0.093 to 0.759, *p* = 0.013). In the multivariable analysis, donor age emerged as a significant predictor of post-transplant complications (HR:1.032, SE = 0.013, Wald = 5.681, df = 1, *p* = 0.017). Results for the Cox proportional hazard risk of rejection after transplantation are presented in Table 3.

## 4. Discussion

To our knowledge, this is the largest long-term retrospective study of keratoconus patients examining risk factors for elevated intraocular pressure after keratoplasty. We noted a high prevalence of glaucoma within this cohort, reaching 41.6%. Additionally, a significant number of patients required surgical intervention, accounting for 48.05%. In the univariable Cox proportional hazards analysis, the following factors demonstrated statistically significant associations (*p* < 0.05) with the risk of glaucoma after transplantation: donor endothelial cell density, single continuous suture, the surgeon performing the surgery, a decrease in the size of the host, a decrease in the size of the donor, and host sex. For many variables, the only factor that remained significant was the surgeon performing the operation (HR = 2.058; 95% CI: 1.410 to 3.004; *p* < 0.001). Within our group, we observed 27 graft failures (14.06%). Among the factors analyzed for risks of post-transplantation failure, significant associations were observed with donor age, time from harvest to transplant, and the surgeon performing the surgery. In the multivariable analysis, donor age emerged as a significant predictor of post-transplant complications (HR = 1.032, SE = 0.013, Wald = 5.681, df = 1, *p* = 0.017). 

Now, we will proceed to a detailed discussion of the results obtained from our study, focusing on a comparative analysis with the findings reported in previously published research.

### 4.1. Incidence and Development Time of Glaucoma

We observed a high incidence of glaucoma in this cohort. We found that in the study group. Among the 185 patients without a history of glaucoma prior to the transplant, 108 (58.40%) did not develop new cases of glaucoma after the transplant, whereas 77 patients (41.6%) did. A similar percentage of patients with increased intraocular pressure after penetrating keratoplasty was recorded in a study [27] conducted at our center between 2009 and 2020, which included 40 eyes (40 patients) after penetrating keratoplasty and 50 eyes (50 patients) after the DALK procedure. A significant difference was observed in the frequency of IOP elevation: 52.5% of eyes after PK surgery and 8% of eyes after DALK. We would like to point out that in our study, covering the years 2014 to 2021, the percentage of patients who underwent lamellar keratoplasty is much lower. This is probably related to the higher average age of patients in our study (48.39 ± 17.6 years) compared to the discussed study (28.6 ± 5.9 years for DALK and 28.4 ± 8.6 years for penetrating keratoplasty). The higher age is associated with a decreasing number and quality of endothelial cells and more frequent cases of Descemet’s membrane rupture, which leads to a higher number of indications for penetrating keratoplasty. The decline in the number of lamellar transplants is also linked to the widespread adoption of cross-linking and the development of increasingly advanced contact lenses. As a result, only patients in the most advanced stages of keratoconus are being considered for PK. In a retrospective study [28], 75 eyes of 64 patients after DALK and 52 eyes of 51 patients after penetrating keratoplasty were included. Increased intraocular pressure was found in 46.2% of eyes after PK and 1.3% after DALK (*p* < 0.001). Secondary glaucoma was detected in 9.6% of patients after PK and in 0% of patients after DALK (*p* = 0.006). The average donor graft size in the PK group was 7.43 ± 1.09 mm. Although the study did not specifically analyze the incidence of glaucoma but rather the increase in intraocular pressure after PK, the result is similar to our reported incidence of glaucoma after transplantation, which was 40.1% (*p* < 0.001). The study described in [29] conducted an analysis including 228 cases after DALK and 274 after PK. Among other factors, the occurrence of postoperative complications in the form of increased intraocular pressure requiring treatment was analyzed. It was found that in the DALK group, increased intraocular pressure affected 11.8% (n = 27) of cases, whereas in the PK group, it occurred in 21.5% of cases (*p* = 0.004). The average donor graft size in the PK group was 8.19 ± 0.19 mm. This result is much lower than that reported in our study. We observed an interesting relationship regarding graft size in the studies discussed. In our study, the average donor graft size was 7.85 ± 0.64 mm. Examining the results from the studies, a noticeable trend emerged: the smaller the average graft size, the higher the percentage of patients with increased intraocular pressure after transplantation or, as in our study, the incidence of glaucoma. Interestingly, in our study, we identified graft size as a statistically significant risk factor for glaucoma after corneal transplantation.

The median time to develop glaucoma after transplantation in our study was 314 ± 67 days. A similar result was obtained in another study [30], where in group I (ectasia, including keratoconus), the mean time for increased intraocular pressure to occur was 6.7 ± 2.6 months.

### 4.2. Risks Factors of Glaucoma

In our study, donor endothelial cell density (ECD/mm^2^) was associated with an increased risk of glaucoma (HR = 0.999; 95% CI: 0.998 to 1.000; *p* = 0.049). It is known that high intraocular pressure and glaucoma are common complications after keratoplasty [31]. In patients undergoing PKP, glaucoma also leads to a greater loss of donor endothelial cells [32]. Although a study [33] stated that the lowest rate of endothelial cell loss was associated with post-keratoplasty patients diagnosed with keratoconus, the significance of the number of endothelial cells as a factor in the development of glaucoma seems very clear to us.

We found that the use of a single continuous suture type was associated with a significantly higher risk of glaucoma (HR = 4.055; 95% CI: 1.503 to 10.936; *p* = 0.006). The significance of suture type in the context of increased intraocular pressure is discussed in a study [34], which indicates that interrupted sutures and a higher number of sutures were associated with increased IOP levels.

We found that a decrease in the size of the host was significantly associated with a higher risk of glaucoma (HR = 0.001; 95% CI: 0.000 to 0.487; *p* = 0.029). Similarly, a decrease in the size of the donor was significantly associated with a higher risk of glaucoma (HR = 0.003; 95% CI: 0.000 to 0.712; *p* = 0.037). Our study showed completely different results compared to those reported in previously published works. The authors of one study [35] showed that a recipient size greater than 8 mm was at higher risk (82%), with an OR of 6.66 (2.45–18.04), for raised intraocular pressure. Another study [36] reported that the incidence of glaucoma was 37% in eyes with 10 mm grafts, 16% in eyes with 8–9.5 mm grafts, and 14% in eyes with 6–7.5 mm grafts. In the conclusions of their article, the authors stated that close-fitting and shallow sutures, along with larger grafts, increase the risk of PKG [37]. None of these studies examined patients after transplantation for keratoconus.

Lastly, female hosts exhibited a significantly lower risk of glaucoma compared to male hosts (HR = 0.292; 95% CI: −0.099 to 0.863; *p* = 0.026). However, it is important to note a limitation of our study: the group of women was considerably smaller than the group of men, which may render any gender-based comparisons somewhat inaccurate.

In the multivariable analysis, the only factor that remained significant was the surgeon performing the surgery. The analysis showed that the surgeon significantly influenced the risk of developing glaucoma (HR = 2.058; 95% CI: 1.410 to 3.004; *p* < 0.001). To our knowledge, no previous study has analyzed the surgeon as a risk factor for glaucoma following keratoplasty, which is undoubtedly a strength of our work. However, it is worth noting that a certain limitation of the study is the uneven distribution of the number and types of surgeries performed by different surgeons. Nonetheless, we propose that the “surgeon factor” may play a role in the development of glaucoma after corneal transplantation.

### 4.3. Graft Failure Rate and Time to Graft Failure

In our group, we observed 27 graft failures (14.06%). The graft failure rate in our study was lower than that reported in a study of 1090 patients, where 21% of grafts failed within five years after penetrating keratoplasty (PK) [38]. It is worth noting that the group in the referenced study consisted of 62% of patients with Fuchs’ dystrophy, which has been shown to have a lower graft survival rate compared to patients with keratoconus [39]. This is likely the primary reason for the observed difference in graft failure rates.

The median time to graft failure in our study was 364 ± 15 days. Previous studies have reported varying mean times to graft rejection after keratoplasty. One study [40] reported a longer time to rejection, with an average of 15.25 ± 14.4 months, while another study [41] reported a shorter time to rejection than ours, with an average of 10.5 ± 9.3 months.

### 4.4. Predictors of Post-Transplant Complications

In our study, donor age was identified as a significant predictor of post-transplant complications (HR = 1.032, SE = 0.013, Wald = 5.681, df = 1, *p* = 0.017). These findings differ from those reported in previous studies. The authors of studies [38,42] found that none of the factors related to tissue processing, such as post-mortem interval, length of storage, endothelial cell density, and donor age, were associated with an increased risk of graft failure when assessing the donor cornea.

Post-transplant glaucoma was not identified as a significant predictor in the occurrence of post-transplant complications. This is an interesting finding, especially in light of previous studies showing that glaucoma and ocular hypertension can exert mechanical stress on the corneal endothelium, exacerbating endothelial cell loss, which is crucial for maintaining corneal transparency and graft viability [43,44]. However, our multivariable analysis indicated that donor endothelial cell density was significantly associated with the development of glaucoma post-transplantation (HR = 0.999, *p* = 0.049), underscoring the complexity of the relationship between these variables.

### 4.5. Summary of the Discussion

We noted a high prevalence of glaucoma within this cohort, reaching 41.6%. We observed that a significant proportion of patients required surgical intervention to manage their elevated IOP, reflecting the severity of glaucoma in this population. Given the association between glaucoma, endothelial cell loss, and graft failure, it is essential to recognize glaucoma as a critical factor contributing to the overall outcome of corneal transplantation.

Although transient IOP elevations in the early postoperative period were effectively controlled in most cases through non-surgical anti-inflammatory therapies, a subset of patients nonetheless developed chronic glaucoma, necessitating more intensive treatment. This progression underscores the importance of rigorous IOP monitoring and timely intervention to maintain graft integrity and function.

### 4.6. Study Limitations

Due to the retrospective nature of the study, there are certain limitations in the methodology used to assess visual acuity. Best-corrected visual acuity was measured by different researchers using various non-standardized charts (such as Snellen type, electronic, or paper). We strongly believe that the methodology for testing visual acuity should be described as accurately and transparently as possible [45].

To conclude the discussion, we would like to explain the decision to analyze all cases within one group. More than 93% of the study group consisted of patients who underwent penetrating keratoplasty (PK), while the remaining less than 7% underwent other types of transplants. Initially, we considered excluding cases involving procedures other than PK. However, during the analysis, we found that the incidence of glaucoma in patients who underwent non-PK procedures (5 out of 13 cases, 38.5%) was similar to that observed in the PK group. Given this similarity and the small number of cases involving procedures other than PK, we decided to include all cases in a single group for analysis.

## 5. Conclusions

The incidence of glaucoma and ocular hypertension in patients with keratoconus is high (41.6%, *p* < 0.001). Several risk factors, including donor endothelial cell density, single continuous suture, the surgeon performing the procedure, reduction in the size of the host cornea, reduction in the size of the donor cornea, and the gender of the host, may contribute to the development of glaucoma after keratoplasty. The surgeon performing the procedure was found to be a statistically significant risk factor for the development of glaucoma. Additionally, donor age emerged as a significant predictor of post-transplant complications.

## Figures and Tables

**Table 1:** Cox proportional hazard of glaucoma after transplantation.

Cox Proportional Hazard of Glaucoma after Transplantation	Significance	Exp (B)	95.0% CI for Exp (B)
			Lower
Donor cell morphology	0.754	1.110	0.579
Donor age	0.714	1.005	0.980
Time from death to harvest	0.477	1.000	0.999
Time from harvest to transplant	0.465	1.054	0.916
Donor ECD/mm^2^	0.049	0.999	0.998
Type of harvest	0.237	0.714	0.409
Donor sex	0.749	0.715	0.092
Type of transplant	0.843	0.939	0.501
Laser assisted transplant	0.204	0.537	0.206
Aprokam	0.395	0.539	0.130
Suture type	0.006	4.055	1.503
Surgeon	<0.001	2.058	1.410
Size of host	0.029	0.001	0.000
Size of donor	0.037	0.003	0.000
Size difference host/donor	0.101	29.217	0.516
Pseudophakia	0.211	0.695	0.393
Host age	0.966	1.001	0.970
Laterality	0.783	1.110	0.527
Host gender	0.026	0.292	0.099

**Table 2:** Frequency distribution of post-transplant complications.

Category	Count	Percent
Rejection	9	4.69
Edema	4	2.08
Persistent erosion	2	1.04
Fungal infection	1	0.52
Bacterial infection	1	0.52
Trauma	2	1.04
Endophthalmitis	3	1.56
Perforation	1	0.52
Bacterial inflammation	1	0.52
Keratomalacia	3	1.56

**Table 3:** Cox proportional hazard risk of rejection after transplantation.

Cox Proportional Hazard Risk of Rejection after Transplantation	Significance	Exp (B)	95.0% CI for Exp (B)
			Lower
Donor cell morphology	0.834	0.849	0.182
Donor age	0.041	1.114	0.979
Time from death to harvest [min]	0.879	1.000	0.997
Time from harvest to transplant [days]	0.083	0.605	0.343
Donor ECD/mm^2^	0.239	1.002	0.999
Type of harvest	0.939	0.922	0.115
Donor sex	0.542	2.342	0.152
Type of transplant	0.10	0.166	0.019
Laser assisted transplant	0.610	4.286	0.016
Aprokam	0.743	0.413	0.002
Suture type	0.605	4.271	0.017
Surgeon	0.013	0.265	0.093
Size of host	0.215	1.121	0.000
Size of donor	0.122	42.057	0.368
Difference size of host/donor	0.372	0.000	0.000
Host age	0.461	1.032	0.948
Laterality	0.802	0.746	0.075
Glaucoma after	0.365	2.691	0.316
Glaucoma treatment	0.558	0.799	0.378
Host gender	0.612	1.555	0.282

## Data Availability

Data are available on request due to restrictions (e.g., privacy or ethical).
